# Macular Hole Surgery Using Gas Tamponade—An Outcome from the Oslo Retrospective Cross-Sectional Study

**DOI:** 10.3390/jcm8050704

**Published:** 2019-05-17

**Authors:** Ingar Stene-Johansen, Ragnheiður Bragadóttir, Beáta Éva Petrovski, Goran Petrovski

**Affiliations:** 1 Center for Eye Research, Department of Ophthalmology, Oslo University Hospital and University of Oslo, Kirkeveien 166, 0450 Oslo, Norway; UXINTE@ous-hf.no (I.S.-J.); UXRADO@ous-hf.no (R.B.); 2 Faculty of Dentistry, University of Oslo, Kirkeveien 166, 0450 Oslo, Norway; beata.petrovski@odont.uio.no

**Keywords:** macular hole, visual acuity, macular hole size, duration of symptoms, ILM peeling, gas tamponade, face-down positioning

## Abstract

**Purpose:** The study aims to determine the anatomical success and functional outcome of pars plana vitrectomy (PPV) for macular holes (MH) performed at a single study center using a consistent procedure of internal limiting membrane (ILM) peeling, SF6 tamponade and 3 days face-down positioning. **Methods:** A retrospective cross-sectional study involving eyes with MHs which underwent 23- or 25-gauge PPV with or without phacoemulsification and all undergoing a 20% SF6 gas tamponade, follow-up to 6 months postoperatively were included at the Department of Ophthalmology, Oslo University Hospital and University of Oslo, Oslo, Norway (12-month study period between 1 January and 31 December 2015) Pre- and post-operative Best-Corrected Visual Acuity (BCVA) assessment, comprehensive eye examination and intraocular pressure (IOP) measurement. as well as Optical Coherence Tomography to determine the diameter of the MH and its closure were all carried out. **Results:** 198 consecutive eyes operated for MH (age: 69.4 ± 7.6 years; 1.6:1 female:male ratio) were included; 35.7%/48.6% had symptoms from 3–6/6–48 months; 5.1% had high-grade myopia, 21.5% focal VMT <1500 µm with or without epiretinal membrane (ERM), and 74.0%/26.0% had phakic/pseudophakic status. Primary closure of the MH occurred in 93.3% of the patients. Lens status and length of symptoms showed no significant correlation with closure of the MH. The pre-operative logMAR visual acuity: 0.8 ± 0.3 (median: 0.7; interquartile range (IQR): 0.5–0.8; range: 0.3–1.7), improved significantly post-operatively: 0.4 ± 0.3 (median: 0.3; interquartile range (IQR): 0.1–0.5; range: −0.02–1.2). BCVA improvement of <0.2, 0.2–0.4 and >0.4 was present in 28.3%, 27.3% and 44.4% of the treated patients. No significant median differences could be detected between the duration of the pre-operative symptoms and the pre-/post-operative visual acuity. Increased IOP was measured in 2.6% of the cases day after surgery. **Conclusions:** Our study found visual outcome not to be dependent upon the length of symptoms in MH patients treated by PPV all undergoing ILM peeling, SF_6_ tamponade and face-down positioning. The large diameter MH was not a limiting factor to achieve improved post-operative BCVA.

## 1. Introduction

The disorders of the vitreo-macular interface (VMI) are relatively common and cause symptoms of metamorphopsia, decreased quality of life and significant loss of vision [1,2]. Macular hole (MH) is a VMI disorder which requires, for most of the cases, an elective vitrectomy [3,4]. The outcome of such surgery depends on the pre-operative visual acuity, duration and size/stage of the hole, presence and grade of myopia, as well as other comorbidities [5,6,7,8,9,10]. In general, MH surgery, improves the pre-to-post-operative visual acuity. Another measurable outcome for successful MH surgery is closure of the hole, which is rather an anatomical and not functional outcome of the surgery. The anatomical success of surgery can vary between 91% and 100%, depending on the different surgical techniques used, as well as the tamponade agents and/or post-operative positioning [11,12,13,14]. In addition to surgery, MH treatment can involve enzymatic vitreolysis with ocriplasmin (Jetrea^®^), in which case an anatomical closure rate of 40% for holes <400 µm in size is expected [15,16], as well as intravitreal gas injection without vitrectomy having anatomical closure rate of approximately 60% for small MH associated with vitreomacular traction [17]. If left untreated, MH can occasionally close on their own.

Vitrectomy increases oxygenation and this in turn increases cataract formation in phakic patients [18]. An increasing number of centers worldwide perform phacovitrectomy surgery in MH patients with cataracts, as well as those over 50 to 65 years of age [19]. Combined surgery appears to be a more cost-effective and safer surgery for such patients, reducing in-hospital and recovery time compared to two separate procedures on two separate occasions [20].

The post-operative complications of vitrectomy for MH can in severe cases include endophthalmitis; other complications are intraocular hemorrhage, post-operative gliosis/fibrosis of the macula, iatrogenic retinal tears and detachment, as well as cataract formation. In vitrectomy alone and in phacovitrectomy procedures, cystoid macular edema can occur in the first 6–12 months after surgery [4,11,17,21,22].

Some MH are associated with epiretinal membranes (ERM) and vitreomacular tractions (VMTs) [1]. Removal of the internal limiting membrane (ILM) together with ERM peeling reduces the risk of tractional forces on the surface of the retina, and reduced the risk of recurrence or non-closure of the MH. Peeling of the ILM is a commonly practiced step in macular surgery [5,6,21].

The present retrospective cross-sectional study from the Department of Ophthalmology, Oslo University Hospital and University of Oslo, Norway, describes the outcome of MH surgery including pre- and post- operative visual acuity, duration and size/stage of the hole, presence and grade of myopia, as well as other comorbidities using a consistent procedure- vitrectomy, ILM peeling, gas (20% SF_6_) and 3 days face-down positioning tamponade performed by all surgeons.

## 2. Methods

This is a retrospective cross-sectional study involving eyes with MHs that underwent 23- or 25-gauge pars plana vitrectomy (PPV) with or without phacoemulsification and all undergoing a 20% SF_6_ gas tamponade at the Department of Ophthalmology, Oslo University Hospital and University of Oslo, Oslo, Norway. Surgeries were carried out over a period of 12 months from 1 January to 31 December 2015. Inclusion criteria included eyes with idiopathic MHs and post-operative follow-up at our or referring offices up to 6 months. Eyes with known pre-operative risks such as ocular trauma, high myopia, previous PPV and co-existing ocular co-morbidity such as AMD, retinal breaks, maculopathy/macular edema, RVO, amblyopia, previous retinal detachment and corneal dystrophy were also included in the study.

Demographic data including age and gender were obtained for each patient as well as duration of symptoms and involved eye. Pre-operative evaluation included Best-Corrected Visual Acuity (BCVA) assessment, slit lamp examination of the anterior segment, IOP measurement and a dilated fundus examination. An MH was diagnosed by slit lamp biomicroscopy with a +90 D lens and confirmed by Optical Coherence Tomography (Nidek RS-3000 Advance, Nidek, Aichi, Japan). The diameter of the macular hole was measured on OCT in all cases.

MH surgery was performed with or without phacoemulsification. Phacoemulsification was performed if a cataract was present. Surgeries were performed under retrobulbar anaesthesia. Phacoemulsification with implantation of an acrylic foldable Intraocular Lens (IOL) in the capsular bag was performed by the same surgeon performing vitrectomy in cases of combined surgery. MH surgeries were performed by more than one surgeon with differences in the surgeons’ experience, while combined surgeries were performed by an experienced surgeon. A PPV with 23- or 25-gauge, standard 3-port approach was done in each case. Central core vitrectomy was performed followed by detachment of the posterior hyaloid using vacuum with the vitrectomy probe. The peripheral vitreous was then removed with careful inspection of the retinal periphery. The macular area was stained with ILM blue dye (DORC) and peeling of the ILM carried out. Where an ERM was present, it was removed before or together with peeling of the ILM. A complete fluid-air-gas (20% SF_6_) exchange was performed and the superonasal and superotemporal cannulae removed, then the conjunctiva was repositioned to cover the sclerotomy sites; if leakage was apparent, the sclerotomies and conjunctiva were sutured accordingly. Consequently, the infusion cannula was then removed and the inferotemporal sclerotomy sealed. Patients were advised to maintain a face-down position for 3 days post-operatively.

After surgery, topical steroids (Maxitrol, 3 times daily for 3 weeks) and cycloplegics (Cyclopentolate 1%, 2 times daily for 10 days) were prescribed. Patients were examined on the first post-operative day 1, at 4 weeks by the local/referring ophthalmologist and up to 6 months post-operatively (see Figure S1 for protocol schematic). IOP was assessed on the first post-operative day. Best-corrected visual acuity when gas was resorbed and IOP were measured at each visit and a slit lamp biomicroscopy was performed to assess the status of the hole. Post-operative complications were documented when present. All the patients had an OCT done pre-operatively to confirm the status of the hole and post-operatively after resorption of the gas. Post-operative visual acuity was assessed up to 6 months post-operatively while anatomical success was defined as hole closure on OCT. The primary outcome measures were anatomical success and visual improvement, while secondary outcome was post-operative complications. At follow-up, MHs were categorized as either open or closed.

The analysis of the data was performed by descriptive statistical analysis; percentage distribution, mean and standard deviation (SD), median and interquartile range (IQR) are shown. Normality of continuous variables was tested on histogram, Q-Q- plot and by Kolmogorov-Smirnov and Shapiro-Wilk tests. Since the normality assumption was not satisfied, Wilcoxon Signed Rank Test was used to compare the median differences of the LogMAR visual acuity before and after surgery for MH, and for the size and duration of symptoms. To compare the mean rank of the pre- and post-operative logMAR between three different groups (sizes of MH and duration of symptoms), Kruskal-Wallis ANOVA with Dunn’s post-hoc test was applied. Best-corrected visual acuity was recorded as a Snellen visual acuity and converted to logMAR for statistical analysis. Chi-square (*χ*^2^) and Fisher’ exact test were used to test the differences of the distribution of categorical variables. Significance limit was set at *p* < 0.05. Statistical Package for STATA (Stata version 14.0; College Station, TX, USA) and SPSS (SPSS version 24, IBM, Armonk, NY, USA) were used for the statistical analyses.

The study adhered to the tenets of the declaration of Helsinki; patient informed consent was obtained prior to surgery and as part of an approved quality and research register in the department.

## 3. Results

The study included 198 consecutive eyes operated for MH (age: 69.4 ± 7.6 years (range: 40–89 years); 61.7% females, which gives 1.6:1 female:male ratio). Eyes with high-grade myopia (larger than −8 D) comprised 5.1%. Focal VMT < 1500 µm was present in 21.5%, and VMT > 1500 µm was detected in any of the eyes. ERM was present in 8.9% of the studied population, and of the later, 3 subjects had both VMT and ERM. A summary of the characteristics of the MHs is shown in Table 1.

From all the subjects, 74.0% and 26.0% were phakic and pseudophakic, respectively. Phacovitrectomy was performed on 26.9% of the phakic patients whose mean age was 72.0 ± 5.8 years (range: 63–85 years). Jetrea^®^ was used only on 2 subjects that were phakic (age: 50 and 69 years) and none of the pseudophakic patients.

All surgeries for MHs in the period of one year were performed with ILM peeling, SF_6_ gas tamponade and face-down positioning post-operatively for 3 days. The MHs closed after the first surgery in 93.3% of the cases. From the unclosed MHs after the first surgery (13 cases), 5 cases had an associated pre-operative disorder such as age-related macular degeneration (AMD), glaucoma, ocular trauma (3 cases), retinal breaks, maculopathy/macular edema (1 case), retinal vein occlusion (RVO), amblyopia, previous retinal detachment and corneal dystrophy (1 case). There were 39 (19.9%) operative complications such as (retinal detachment (1 case), vitreal hemorrhage (5 cases), incomplete posterior vitreous detachment (PVD) (3 cases), lens damage during surgery (1 case), retinal break (24 cases) and higher intraocular pressure on the first post-operative day (5 cases). Post-operative complications occurred in 25 cases (12.8%) such as cystoid macular edema (2 cases) and cataract (23 cases). The incidence of complications during and after surgery was overall higher with lesser surgeon experience. Presence of own lens or pseudophakia and length of symptoms showed no significant correlation with closure of the MH (*P* = 0.794 and 0.566, respectively).

**Figure 1:**
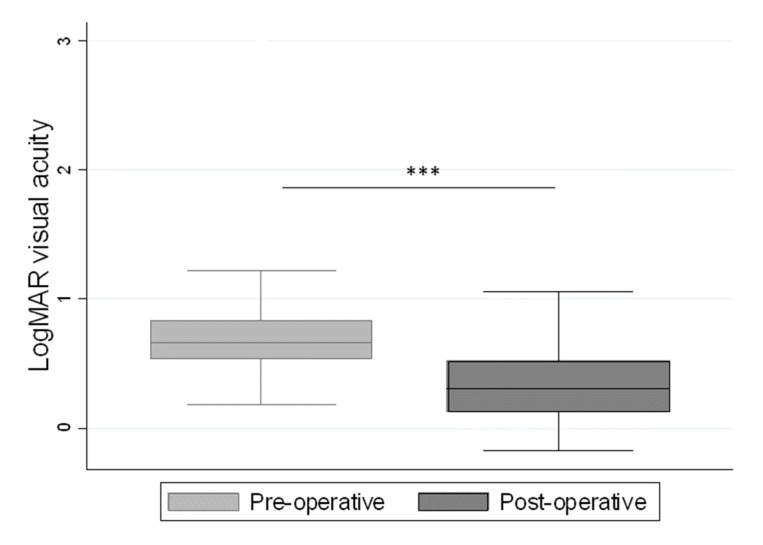
LogMAR visual acuity before and after surgery for macular hole. *** *p* < 0.001

The pre-operative logMAR visual acuity was 0.8 ± 0.3 (median: 0.7; interquartile range (IQR): 0.5–0.8; range: 0.3–1.7), while the post-operative logMAR visual acuity improved significantly to 0.4 ± 0.3 (median: 0.3; interquartile range (IQR): 0.1–0.5; range: −0.02–1.2) (Figure 1).

**Figure 2:**
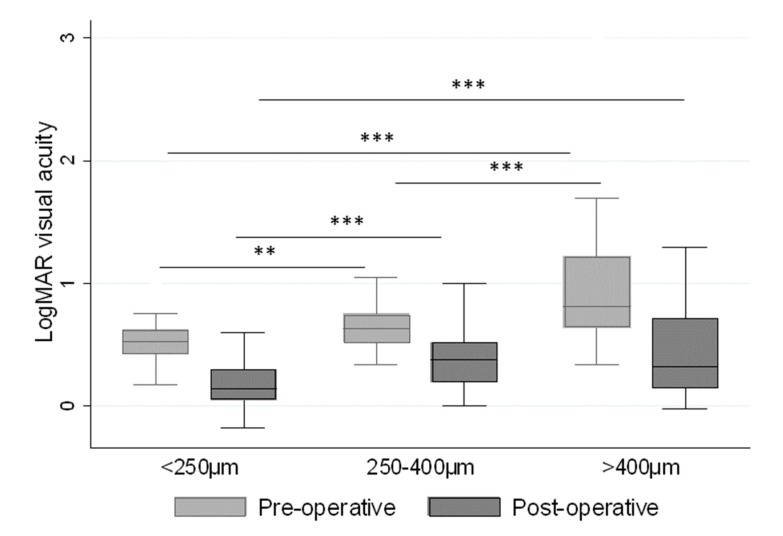
The relationship between pre- and post-operative LogMAR visual acuity and the size of the macular hole. *** *p* < 0.001, ** *p* < 0.01.

There was significant median difference between the size of the MH (<250 µm, 250–400 µm and >400 µm) and the pre-operative visual acuity, with the logMAR improvement in visual acuity shown in Figure 2 for each size group. These median differences could be detected post-operatively between the MH sizes <250 µm and 250–400 µm, and the <250 µm and >400 µm groups.

The distribution of the visual acuity and its improvement post-operatively on logMAR by less than 0.2 (e.g., 2 lines or 10 letter improvement) was present in 28.3% of the patients, between 0.2 and 0.4 (e.g., 2–4 lines or 10–20 letters improvement) in 27.3%, and by more than 0.4 (e.g., more than 4 lines or 20 letters improvement) in 44.4% of the patients (Table 2).

**Figure 3:**
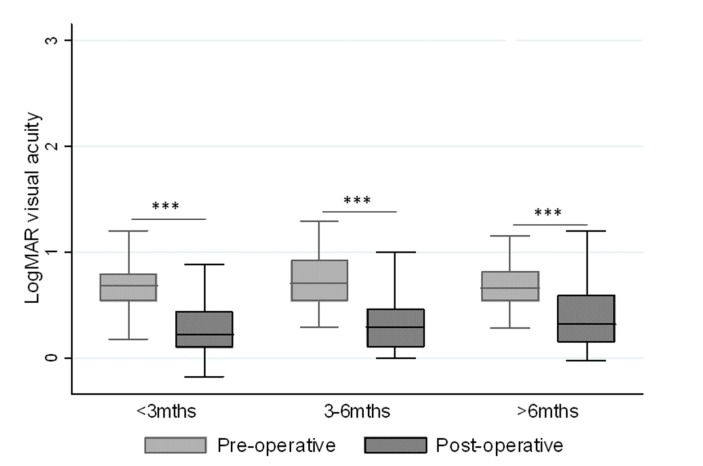
The relationship between pre- and post-operative logMAR visual acuity and the duration of the pre-operative symptoms. *** *p* < 0.001.

Statistically significant median differences occurred between the pre-and post-operative visual acuity for all symptom durations (Figure 3), while no statistically significant median differences could be detected between the duration of pre-operative symptoms (<3, 3–6 or >6 months) and the post-operative visual acuity. Reoperation was performed on 5.2% of the cases in which the post-operative visual acuity remained similar to that pre-operatively.

## 4. Discussion

The diseases of the vitreo-macular interface (VMI) affect the posterior vitreous cortex, the ILM and the intervening extracellular matrix [2]. Their classification, including that of MHs, has been made by the International Vitreomacular Traction Study Group [1].

Incomplete PVD with persistent attachment in the macular region can cause an anomalous traction to the structures of the posterior pole. Anatomic variations such as myopia/hypermetropia, trauma, previous surgery, have all been shown to increase the risk for VMI disorders [23]. In our study, only 5.1% of the patients had high-grade myopia,

MHs, although with low prevalence (Beijing Eye Study: 0.09 ± 3.04% [24]), have significant impact on the quality of life and visual acuity of the elderly population, having higher preponderance in females, as found in our study as well (1.6:1). Tangential and/or anterior-posterior traction cause cystoid spaces in the inner retinal layers which can progress to the outer retinal layers, and vitreous adherence at the fovea and optic disc are few of the causes for MH formation [2,9,10,25].

Indications for surgery of the different stages or sizes of MHs have been described extensively by others [3,4,5,26]. In this study, the surgery of choice for MHs was 23- or 25-gauge PPV with or without cataract surgery. Gas tamponade for treatment of MHs with PPV has been used since the year 1991 [22]. Different gas compositions and concentrations, as well as use of air, with various duration of the face-down position after surgery have been used and reported extensively [13,25,26,27,28,29,30,31,32,33]. Although there are reports on the face-down posturing giving no improvement to outcomes, the present study had consistent face-down posturing for 3 days post-operatively for all sizes of MHs. The rationale we used for the gas (SF_6_) tamponade is the waterproofing gas effect upon the MH from the vitreous cavity or the surface-tension and mechanical effect at the gas-liquid interface [34]. The sutureless PPV was assumed to cause close to nil gas leakage after surgery, or when leakage was apparent, suturing of the sclerotomies assured avoidance of insufficient tamponade in all cases [35].

ILM peeling of the macula was performed on all surgeries included in the study, and no ILM-flap technique was used—all cases achieved a significantly better post-operative visual acuity (logMAR 0.4) [36], which was similar or better than the visual acuity reported by Kannan NB et al. [7] and Kim et al. [8]. Better BCVA after MH surgery has been reported by several other studies as well [8,19,21,27]. The pre-operative BCVA has been considered to be the most important prognostic factor for improved visual acuity post-operatively, as well as for retained better visual acuity [6,9]. In our study, close to half of the patients operated for MH achieved improvement in visual acuity post-operatively of more than 20 letters or 4 lines. Furthermore, the size (<400 µm) and duration of symptoms (<6 months) have been considered to be important prognostic factors for better anatomical and visual outcomes [37,38]. Accordingly, we found significant correlation between the size of the MH (<250 µm, between 250–400 µm, and ≥400 µm) and the pre- and post-operative visual acuity, and interestingly, found no significant correlation in the patient group which had more than 6 months of pre-operative symptoms.

Increased IOP was measured in 2.6% of the cases, which is much lower compared to 20–21% reported by others on the first post-operative day [39]; the IOP could be regulated with anti-glaucoma agents. SF_6_ belongs to a group of expansile gases used as tamponade agents, and its concentration can vary in different studies [7,40]; however, our study used in all cases a 20% SF_6_, which is considered non-expansile concentration. Thus, the incidence of increased IOP post-operatively could be much lower or near to those studies reporting no IOP spike post-operatively [41]. The risk for post-operative hypotony reported to occur with 23G vitrectomy [10,42,43] was not apparent in the present study. Besides increased IOP, other post-operative complications can occur, such as cataract formation, visual field defects, retinal breaks and risk for detachment, as well as proliferative vitreoretinopathy [34]. Our study as well had some of these complications, and their appearance seemed to decrease with increased experience of the surgeon. The complication rate of senior and junior surgeons varied between 8.7–22.2% and 26.7–35.0%, respectively, with one junior surgeon having performed single surgery which had complications.

Cataract formation after PPV, in particular with air or gas tamponade is a well-known side effect of the surgery; a study reported up to an 81% cataract risk after 6 months, 98 and 100% risk at 1 and 2 years, respectively, post-vitrectomy [44]. For that reason, and due to the presence of a certain level of cataract, a quarter of our phakic patients underwent phacovitrectomy procedure, followed by the standard face-down positioning.

The use of SF_6_ after fluid-air exchange is simple, requires no additional intravitreal injection of concentrated gas through a pars plana injection site, but instead uses the surgical trochars for delivery.

The advantages of the present retrospective cross-sectional study are its consistent use of ILM peeling, gas tamponade of same concentration and post-operative face-down (3 days) positioning after a 23- or 25-gauge PPV.

Our study found that the visual outcome is not dependent on the length of symptoms in the studied population and that the presence of mostly large MH still did not affect the post-operative visual acuity improvement with the procedure of choice.

## Figures and Tables

**Table 1:** Macular hole size and duration of symptoms.

**Size of the Macular Hole**	**Percent of Subjects**
Small (<250 µm)	20.2%
Medium (between 250–400 µm)	37.3%
Large (≥400 µm)	42.5%
**Duration of Symptoms**	**Percent of Subjects**
≤3 months	15.7%
3–6 months	35.7%
>6 up to 48 months	48.6%

**Table 2:** Distribution of the logMAR difference pre- and post- operatively.

LogMAR Difference Pre- and Post-Operatively	<0.2	0.2–0.4	>0.4
*N* (%)	56 (28.3%)	54 (27.3%)	88 (44.4%)

## Supplementary Materials

The following are available online at https://www.mdpi.com/2077-0383/8/5/704/s1, Figure S1: Schematic of the steps used in the treatment protocol.
