# Supplementary figures and images for: Macular Hole Surgery Using Gas Tamponade—An Outcome from the Oslo Retrospective Cross-Sectional Study
Source: J Clin Med. 2019 May 17;8(5):704. doi: 10.3390/jcm8050704 (PMC6572686; doi:10.3390/jcm8050704)

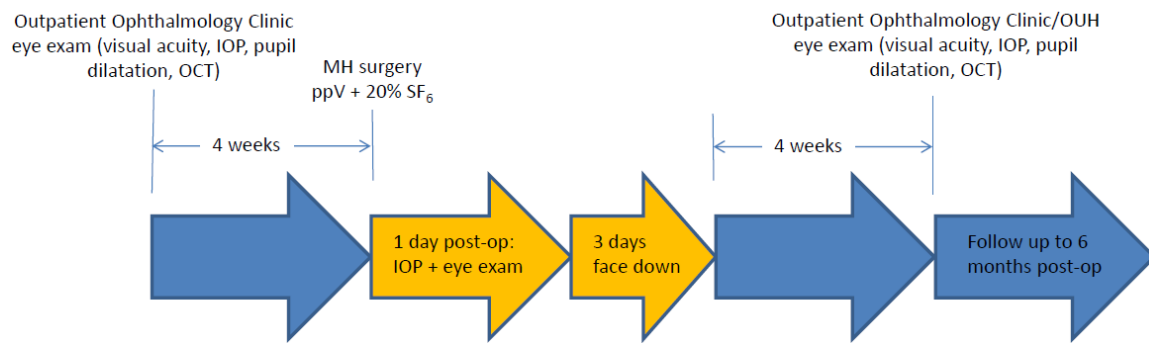

**Figure S1.** Schematic of the steps used in the treatment protocol.

Supplement: Supplementary file 1 [file jcm-08-00704-s001.pdf]
